# Visibility of Board-Certified Dermatologists on TikTok

**DOI:** 10.2196/46085

**Published:** 2024-01-05

**Authors:** Chaitra Subramanyam, Alyssa Becker, Julianne Rizzo, Najiba Afzal, Yvonne Nong, Raja Sivamani

**Affiliations:** 1 College of Osteopathic Medicine of the Pacific Northwest Western University of Health Sciences Lebanon, OR United States; 2 John A Burns School of Medicine University of Hawaii at Manoa Honolulu, HI United States; 3 College of Medicine University of California Davis Sacramento, CA United States; 4 College of Human Medicine Michigan State University Flint, MI United States; 5 Pacific Skin Institute Sacramento, CA United States

**Keywords:** board, certification, board certification, health, media, public, social, TikTok, social media, health information, misinformation, diagnosis, users, medical training, training, media content, skin, derma, derm, dermatologist, dermatology, epidermis, dermatitis, cellulitis, skin doctor, skin, hair, nail

## Abstract

Tik Tok is an emerging social media platform that provides a novel opportunity for health practitioners such as dermatologists to disseminate accurate health information.

## Introduction

TikTok is a video-sharing social media platform with over 1.1 billion active users since its launch in 2016 [1]. Social media platforms such as TikTok are used by medical and nonmedical professionals to share health information. However, health misinformation spreads more quickly than evidence-based information, posing a public health issue [2]. Our study aimed to categorize popular dermatology-related posts and analyze the visibility of board-certified dermatologists (BCD) on TikTok.

## Methods

The methods were designed based on a previous study that examined dermatology content on Instagram by Park et al [3]. First, a list of top dermatologic diagnoses and procedures was compiled based on the National Ambulatory Medical Care Survey and the American Society of Dermatologic Survey of Dermatologic Procedures [4,5]. Then, all of the terms were queried as hashtags in TikTok’s search feature on January 2, 2021.

The 20 dermatologic conditions and procedures with the highest total views were identified. Profession-specific hashtags (#dermatology, #boardcertifieddermatologist, #dermatologist, and #derm) were also queried. The term with the highest total views was chosen among synonymous terms.

The first 10 posts under each of the 44 hashtags were then viewed. Top posts were selected through TikTok’s private algorithm, which uses total views, followers, and other metrics. Users’ self-reported occupations were identified, and board certifications were confirmed through the Certification Matters website [6]. Posts were categorized into 4 categories: educational, self-promotional, non–paid product placements, and advertisements. Educational content was identified as any post that aimed to provide informative material regarding a dermatologic condition and/or procedure. Self-promotional content was defined as posts intended to advance the user’s professional pursuits. Non–dermatology-related posts were excluded.

## Results

Of the 18.68 billion total views of the hashtags investigated, 12.9 billion (69.1%) were related to skin conditions, 4.26 billion (22.8%) were related to dermatologic procedures, and 1.52 billion (8.17%) were profession-specific.

Out of 231 unique user profiles that accounted for the 360 top dermatology-related posts, 70 (30.3%) were patients, 66 (28.57%) were medical professionals, and 11 (4.76%) were estheticians (Table 1).

BCD and dermatology residents made up 15 (6.49%) and 7 (3.03%) of the top dermatology-related content creators, respectively. In the queried hashtags, verified BCD and dermatology residents created 13.89% (50/360) and 8.89% (32/360) of the top posts, respectively.

Of the identified top posts, 46.67% (168/360) were educational, 27.50% (99/360) were self-promotional, 13.89% (50/360) were non–paid product placements, and 0.83% (3/360) were advertisements.

A total of 29.76% (50/168) and 70.24% (118/168) of educational posts were created by nonmedical and medical professionals, respectively; specifically, BCD created 20.83% (35/168) and dermatology residents created 18.45% (31/168). BCD were responsible for only 30% of the profession-specific hashtag-identified posts (Table 2).

**Table 1:** Medical professionals versus nonmedical professionals who created top dermatology-related TikTok videos (total unique creators: N=231).

Category		Self-identified, n (%)	Residency or board-certified status confirmed, n (%) of total unique creators
**Medical professionals**			
	Dermatologists	15 (6.49)	13 (5.63)
	Dermatology residents	7 (3.03)	7 (3.03)
	Physicians in other specialties	21 (9.09)	16 (6.93)
	Nurse practitioners	6 (2.6)	4 (1.73)
	Physician’s assistants or associates	2 (0.87)	2 (0.87)
	Registered nurses	4 (1.73)	2 (0.87)
	Unspecified	11 (4.76)	0 (0)
	All medical professionals	66 (28.57)	44 (19.05)
**Nonmedical professionals**			
	Patients	70 (30.3)	N/A^a^
	Estheticians	11 (4.76)	N/A
	Verified account (brand or influencer)	12 (5.19)	N/A
	Other	72 (31.17)	N/A
	All nonmedical professionals	165 (71.43)	N/A

^a^N/A: not applicable.

**Table 2:** Users responsible for the top 10 videos under each profession-specific hashtag.

Users	Hashtag, n					Total, n (%)
	#dermatology	#derm	#dermatologist	#boardcertifieddermatologist		
Board-certified dermatologist	2	1	1	8	12 (30)	
Dermatology resident	4	0	8	1	13 (32.5)	
Internal medicine physician	0	7	0	0	7 (17.5)	
Registered nurse	1	0	0	1	2 (5)	
Esthetician	0	1	0	0	1 (2.5)	
Other	3	1	1	0	5 (12.5)	

## Discussion

Our results suggest that most of the popular dermatology-related content on TikTok is created by individuals without verifiable medical training. This highlights a space for BCD to showcase their profession and prevent the spread of health misinformation. As the use of social media platforms like TikTok continues to grow, BCD have an opportunity to increase their presence as a credible source for the public to acquire dermatologic knowledge.

The use of hashtags explicitly related to dermatology by users who are not BCD or dermatology residents may mislead TikTok users. Transparency regarding professional health care credentials on TikTok may improve credibility. There is currently no way to verify professional credentials on TikTok; a feature to distinguish medical professionals from nonmedical professionals can add to the visibility of BCD and help users make informed decisions regarding their source of health information online.

## Abbreviations

BCD: board-certified dermatologists

## References

[1] O'Sullivan NJ, Nason G, Manecksha RP, O'Kelly F (2022). The unintentional spread of misinformation on 'TikTok'; a paediatric urological perspective. J Pediatr Urol.

[2] Vosoughi S, Roy D, Aral S (2018). The spread of true and false news online. Science.

[3] Park JH, Christman MP, Linos E, Rieder EA (2018). Dermatology on Instagram: an analysis of hashtags. J Drugs Dermatol.

[4] Wilmer EN, Gustafson CJ, Ahn CS, Davis SA, Feldman SR, Huang WW (2014). Most common dermatologic conditions encountered by dermatologists and nondermatologists. Cutis.

[5] (2019). ASDS members performed more than 12.5 million treatments in 2018. American Society for Dermatologic Surgery.

[6] Certification Matters.

